# Diabetic Cardiovascular Disease Induced by Oxidative Stress

**DOI:** 10.3390/ijms161025234

**Published:** 2015-10-23

**Authors:** Yosuke Kayama, Uwe Raaz, Ann Jagger, Matti Adam, Isabel N. Schellinger, Masaya Sakamoto, Hirofumi Suzuki, Kensuke Toyama, Joshua M. Spin, Philip S. Tsao

**Affiliations:** 1 Division of Cardiovascular Medicine, Stanford University School of Medicine, Stanford University, Stanford, CA 94305, USA; E-Mails: kayama@jikei.ac.jp (Y.K.); uwe.raaz@googlemail.com (U.R.); jagger.ann@gmail.com (A.J.); matti.adam@uk-koeln.de (M.A.); isabelschellinger@googlemail.com (I.N.S.); ktoyama@stanford.edu (K.T.); josh.spin@gmail.com (J.M.S.); 2 VA Palo Alto Health Care System, Palo Alto, CA 94304, USA; 3 Division of Diabetes, Metabolism and Endocrinology, Department of Internal Medicine, Jikei University School of Medicine, 3-25-8 Nishi-Shinbashi, Minatoku, Tokyo 105-0003, Japan; E-Mails: m-sakamoto@umin.ac.jp (M.S.); hiro1020@jikei.ac.jp (H.S.)

**Keywords:** diabetes mellitus, oxidative stress, cardiovascular disease, diabetic heart, diabetic vascular disease

## Abstract

Cardiovascular disease (CVD) is the leading cause of morbidity and mortality among patients with diabetes mellitus (DM). DM can lead to multiple cardiovascular complications, including coronary artery disease (CAD), cardiac hypertrophy, and heart failure (HF). HF represents one of the most common causes of death in patients with DM and results from DM-induced CAD and diabetic cardiomyopathy. Oxidative stress is closely associated with the pathogenesis of DM and results from overproduction of reactive oxygen species (ROS). ROS overproduction is associated with hyperglycemia and metabolic disorders, such as impaired antioxidant function in conjunction with impaired antioxidant activity. Long-term exposure to oxidative stress in DM induces chronic inflammation and fibrosis in a range of tissues, leading to formation and progression of disease states in these tissues. Indeed, markers for oxidative stress are overexpressed in patients with DM, suggesting that increased ROS may be primarily responsible for the development of diabetic complications. Therefore, an understanding of the pathophysiological mechanisms mediated by oxidative stress is crucial to the prevention and treatment of diabetes-induced CVD. The current review focuses on the relationship between diabetes-induced CVD and oxidative stress, while highlighting the latest insights into this relationship from findings on diabetic heart and vascular disease.

## 1. Introduction

The worldwide incidence of diabetes mellitus (DM) has recently increased rapidly due to lifestyle changes, with DM projected to affect over 300 million people by 2015 [1]. DM is associated with a wide array of complications, and the associated decrease in quality of life (QOL) of affected patients, thus contributing to the staggering increase in healthcare expenditure. DM is thought to cause not only microangiopathy (associated with the three major diabetic complications, namely diabetic retinopathy, nephropathy and neuropathy) but to constitute a major risk factor for macroangiopathy, such as coronary artery disease (CAD) and cerebrovascular disease [2,3].

The Framingham and MERIT studies demonstrate that patients with DM are two to four times more likely to develop CVD, and have three times higher overall mortality rate compared to those without DM [4,5,6]. It is also shown that the risk for HF is increased in DM patients even after adjustment for CAD and hypertension [5].

It is also known that oxidative stress and inflammation play a key role in the pathogenesis and progression of diabetes-induced CVD, where the increased expression of inflammatory proteins or cytokines such as C-reactive protein (CRP) or oxidative stress-related proteins is shown to serve as a biomarker for the onset of CVD and HF [7]. Thus, it is of great concern that CVD is far more common in patients with DM than the non-diabetic population. Furthermore, oxidative stress is reported to be increased in patients with DM and animal models of DM, with increases noted in reactive oxygen species (ROS) in rat models of DM [8], as well as in 8-hydroxy-deoxyguanosine (8-OHdG) as a marker of DNA oxidation disorder, 8-*iso*-prostaglandin F2a resulting from arachidonic acid peroxidization in blood and urine, and serum peroxidized fat and oxidized low-density lipoproteins (LDL) in patients with DM [9,10,11,12].

It is also recently shown that pancreatic β-cell loss is characteristic of both type 1 and 2 DM and is primarily due to cell damage and death resulting from increased inflammation and oxidative stress at the tissue level [13,14].

Again, oxidative stress is thought to be a major factor contributing to the development and progression of diabetic complications, which is also closely associated with insulin resistance and impaired insulin secretion resulting in the development of DM. Thus, oxidative stress in DM constitutes an important factor implicated not only in the development of diabetic complications but also in the development of DM itself.

The present review provides an overview of oxidative stress followed by a discussion of oxidative stress in relation to the diabetic heart or diabetic heart disease (DHD) and diabetic vascular disease (DVD) as diabetic cardiovascular complications.

## 2. Oxidative Stress: An Overview

Oxidative stress is defined as a state in which ROS overproduction *in vivo* exceeds the buffering capacity of antioxidant enzymes and antioxidants thus resulting in a local imbalance between ROS production and destruction. When available in appropriate amounts, ROS are shown to act as signal transduction molecules providing cell protection, while, in contrast, when available in large excess, they are shown to modify/degenerate biological macromolecules, e.g., nucleic acid (DNA degeneration), lipids (lipid oxidation), and proteins (membrane protein degeneration), thus inducing cell dysfunction or death.

**Figure 1:**
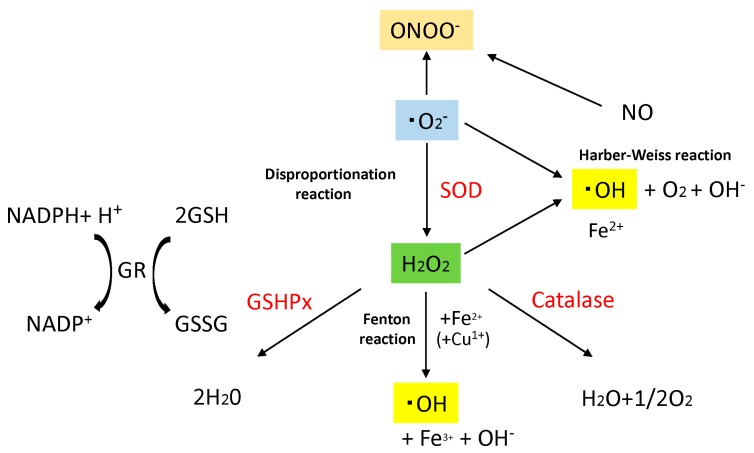
Major generative and eliminative reaction of reactive oxygen species (ROS). Sequential reduction of oxygen resulting in ROS generation, and major ROS generative reaction and eliminative reaction are shown. Superoxide dismutase (SOD) catalyzes the dismutation of superoxide (·O_2_^−^) into H_2_O_2_ and O_2_. Catalase dismutates H_2_O_2_ into water and molecular oxygen. Glutathione peroxidase (GSHPx) eliminates H_2_O_2_ by using GR for another substrate, and generates water. Disproportionation reaction, Harber–Weiss reaction and Fenton reaction are shown. Superoxide anion (·O_2_^−^), hydrogen peroxide (H_2_O_2_), hydroxyl radical (·OH), Peroxynitrite (ONOO^−^), Superoxide dismutase (SOD), Glutathione peroxidase (GSHPx), Glutathione (GSH), Glutathione–S–S–Glutathione (GSSG), Glutathione Reductase (GR), Nicotinamide adenine dinucleotide phosphate (NADPH), Nitric Oxide (NO).

ROS are a group of short-lived, low-molecular-weight compounds derived from oxygen inhaled by aerobic respiration and through a variety of reactions oxygen undergoes *in vivo*, and include superoxide anion (·O_2_^−^), hydrogen peroxide (H_2_O_2_), hydroxyl radical (·OH^−^), and peroxynitrite (ONOO^−^) (Figure 1). While ROS are being constantly produced *in vivo*, normally, their production is regulated to remain below a certain level by antioxidant enzymes *in vivo*, such as superoxide dismutase (SOD), glutathione peroxidase (GSHPx) or catalase (Figure 1) or by antioxidants, such as glutathione and ascorbic acid (vitamin C), In contrast certain exposures, such as ultraviolet (UV) radiation, chemicals, or inflammatory cytokines lead to increased intracellular ROS. Excess ROS production, that exceeds the buffering capacity of antioxidant enzymes and antioxidants, tips the balance toward a more oxidative state.

Sources of ROS production in the human tissue include the mitochondrial electron transport system, NADPH oxidases, xanthine oxidase, uncoupled nitric oxide synthase (NOS), and arachidonic acid metabolism pathways (12/15 lipoxygenase), but they vary in their pathological role and importance depend on the disease and the organ.

**Figure 2:**
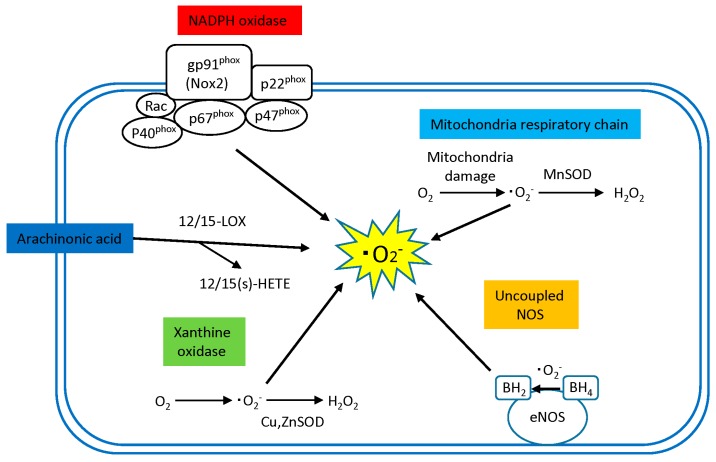
Sources of ROS in the diabetic heart. NADPH oxidase, mitochondria respiratory chain, Arachidonic acid (AA), xanthine oxidase and uncoupling of NOS are major source of ROS in the diabetic heat. Activated NADPH, dysfunctional mitochondrial respiratory chain, decreased availability of tetrahydrobiopterin (BH_4_) in uncoupled eNOS, activated 12/15-LOX pathway in AA and xanthine oxidase generate superoxide anion (·O_2_^−^) in the heart. SOD isoforms, MnSOD, and CuZn SOD dismutate superoxide anion (·O_2_^−^) to produce hydrogen peroxide (H_2_O_2_). Superoxide anion (·O_2_^−^), hydrogen peroxide (H_2_O_2_), hydroxyl radical (·OH), endothelial nitric oxide synthase (eNOS), superoxide dismutase (SOD), dihydrobiopterin (BH_2_), tetrahydrobiopterin (BH_4_).

Of these, the mitochondrial electron transport chain, NADPH oxidase, and xanthine oxidase are thought to be primary sources of ROS production in cardiomyocytes [15], while NADPH oxidase, xanthine oxidase, and uncoupled NOS are thought to be primary sources of ROS production in vascular cells [16] (Figure 2).

### 2.1. Sources of ROS Production in Diabetic Cardiovascular Disease

#### 2.1.1. Mitochondrial Electron Chain

Mitochondria are intracellular organelles accounting for a greater part of the energy required for cell survival through oxidative phosphorylation. In oxidative phosphorylation, superoxide is produced at a rate of 1% to 5% of all oxygen being consumed, even under physiological conditions. Under normal conditions, superoxide becomes metabolized and detoxified through the action of SOD or catalase located in mitochondria or cellular cytoplasm. Of note, it is suggested that mitochondrial ROS are a major cause of oxidative stress associated with DM [17]. *In vitro*, hyperglycemia in vascular endothelial cells—simulated by culturing cells under high glucose concentration—leads to increased ROS. This increase can be suppressed by complex II inhibitors and uncoupling agents in the mitochondrial electron transport chain. Furthermore, suppressing ROS through either of these pathways, reduce PKC activity, NF-κB activity, and advanced glycation end product (AGE) production [17]. Various metabolic abnormalities have been suggested to help explain the increased ROS including AGE production, glucose autoxidation, impaired polyol metabolism, xanthine oxidase activation, increased hexosamine metabolism, increased mitochondria-derived ROS production and NAD (P) H oxidase activation via activation of the PKC pathway [18]. Moreover, studies have shown that mitochondrial ROS not only result from but also cause these intracellular abnormalities.

As intracellular organelles involved in ATP synthesis (oxidative phosphorylation), mitochondria abound in cardiomyocytes. Once oxygen molecules (O_2_) are taken inside the body, electrons are removed and transferred by the inner mitochondrial membrane electron transport chain to O_2_ to form H_2_O. During this process, a proportion of O_2_ is reduced to ·O_2_^−^, which interacts with SOD and becomes metabolized into H_2_O_2_, which, in turn, interacts with intracellular Fe^2+^ (Fenton reaction) or Cu^1+^ to form more oxidative hydroxyl radical (·OH^−^), which, although short-lived, is known to promote oxidative degeneration of nucleic acids, lipids and cytoskeleton proteins (Figure 1).

To date, numerous reports described the role of ROS in cardiomyocytes as produced through the mitochondrial electron transport system. Ide *et al.* documented enhanced cardiomyocyte mitochondrial ·O_2_^−^ in the failing myocardium using electron spin resonance spectroscopy, suggesting that cardiomyocyte mitochondria constitute a major source of ROS production in HF [19]. In addition, ROS production associated with disruption of the mitochondrial electron transport system plays a key role in the pathogenesis of ischemia/reperfusion states [20].

#### 2.1.2. NADPH Oxidases

NADPH oxidase is primarily an ROS-generating enzyme in professional phagocytes (*i.e*., neutrophils, monocytes and macrophages), which becomes reactivated and mediates bactericidal activity against invading microorganisms, thus playing an extremely important role in the mechanisms of host defense against infectious agents [21]. Additionally, NADPH oxidase-generated ROS are also known to serve as intracellular signaling molecules implicated in intracellular processes including synthesis of thyroid hormones [21,22].

**Figure 3:**
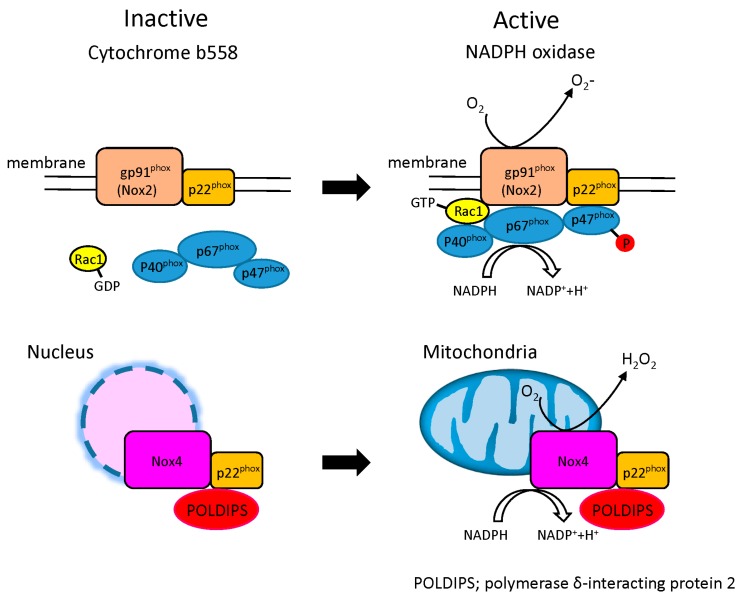
Structure of NADPH oxidase in the heart. NADPH oxidase complex is composed of two major components. Plasma membrane spanning cytochrome b558 composed of p22phox and a Nox subunit (gp91phox (Nox2), Nox4) and cytosolic components composed of four regulatory subunits (p47 phox, p67 phox, p40 phox and Rac1). The low molecular weight G protein rac1 participates in assembly of the active complex. Upon activation, cytosolic components interact with cytochrome b558 to form an active NADPH oxidase enzyme complex, resulting in release of ·O_2_^−^. The primary Nox subunit isoforms in cardiac cells are Nox2 and Nox4. Nox4 oxidase localizes intracellular organelles around the nucleus. The activity of Nox4 results in the direct release of hydrogen peroxide (H_2_O_2_) in mitochondria. The mechanisms underlying the generation of hydrogen peroxide by Nox4 oxidase are yet to be fully characterized.

Known as a member of the Nox family, NADPH oxidase is a multi-molecular enzyme composed of plasma membrane spanning cytochrome b558 (p22phox, gp91phox (Nox2)) and cytosolic components (rac, p47phox, p67phox, p40phox). While this pathway is usually in a resting state, it becomes activated against invading microorganisms and promotes translocation of its cytosolic components to the plasma membrane to form an active NADPH complex to allow transfer of electrons to O_2_ to generate ·O_2_^−^ (NADPH + 2O_2_ → NADP+ 2·O_2_^−^ + H^+^) (Figure 3).

The major function of NADPH oxidase is to generate ROS, which sets it apart from other pathways that merely produce ROS as a byproduct. While primarily thought of as a phagocyte ROS-producing enzyme, NADPH has also been known to largely account for ROS production in atherosclerotic vascular smooth muscle cells (VSMC). More recent data suggest that NADPH is a primary ROS-producer not only for VSMC, but also for other cardiovascular cells such as cardiomyocytes, vascular endothelial cells and adventitial fibroblasts [22,23].

There are seven members of the Nox family (Nox1–5) and dual oxidase (Duox1–2) that have been identified, with Nox2 and Nox4 as the major myocardial isoforms. These have different cellular localizations, with Nox2 localized to the cell membrane, Nox4 to intracellular organelles around the nucleus. The intracellular localization reflects differences in their physiological, and by consequence pathological, properties. Nox4 particularly has a mitochondrial localization signal, and is expressed predominantly in the mitochondria of cardiac muscle cells [24] (Figure 3).

More recently, it has been reported that the expression of Nox4 and the production of ROS are increased in pressure overloaded hearts [25]. Disruption of Nox4 in cardiomyocyte significantly reduced cardiac hypertrophy, interstitial fibrosis and cardiomyocyte apoptosis in the presence of pressure overload, thereby improving cardiac dysfunction and reduced mitochondrial dysfunction [25]. Moreover, infarct size after ischemia/reperfusion was also reduced in cardiomyocyte-specific Nox4 deletion mice compared to those of wild-type mice [26].

Several reports have also demonstrated increased activity of NADPH oxidase and expression of Nox4 is increased in cardiomyocytes exposed to high glucose [27] as well as enhanced ROS production by NADPH oxidase in the heart of diabetic mouse models [28,29]. Furthermore, myocardial hypertrophy and fibrosis in type 1 DM is characterized by increased expression of Nox1 and Nox2 [30,31]. Similar increases in Nox have also been described in models of type 2 DM [32]. Moreover, in the murine model if STZ-induced DM, a reduction in NADPH oxidase activation, due to Rac1 deficiency, has been shown to have beneficial effects upon myocardial remodeling [33]. Taken together, these data suggest that NAPDH-generated ROS is associated with multiple cardiovascular complications in DM.

#### 2.1.3. Xanthine Oxidase

Xanthine oxidase is an enzyme present in the cytoplasm and catalyzes oxidation of its substrates hypoxanthine and xanthine to uric acid using O_2_ as an electron receptor and, in the process, produces ·O_2_^−^ and H_2_O_2_. These ROS are usually eliminated by antioxidant enzymes abundantly present in the cytoplasm (e.g., Cu, Zn-SOD, GPx) (Figure 2). However, hypoxanthine and xanthine oxidase are shown to react acutely with O_2_ in ischemia/reperfusion states to produce a large amount of ·O_2_^−^ and H_2_O_2_, thus inducing cell damage as a consequence.

While this pathway is known to usually serve as an important source of ROS production in vascular endothelial cells, a similar role has been suggested for the xanthine oxidase pathway in cardiomyocytes as well [34]. Administration of the xanthine oxidase inhibitor, allopurinol, leads to improvements in cardiac as well as vascular function in a canine model of tachyarrhythmia-prone HF [35].

#### 2.1.4. Arachidonic Acid (12/15 Lipoxygenase)

Lipoxygenases (LOX) constitute another important source of ROS production in both cardiomyocytes and vascular cells. LOX are a family of lipid-peroxidizing enzymes that oxidize free and esterified polyenoic fatty acids to form the corresponding hydroperoxy derivatives [36]. 12/15-LOX is a member of the LOX family that catalyzes the step from arachidonic acid (AA) to 12(*S*)-hydroxyeicosatetraenoic acids (12(S)-HETE) and 15(S)-HETE (Figure 2). Interestingly, while LOX metabolites of AA mediate angiotensin II stimulation of NAD(P)H oxidase in VSMC [37], AA metabolism is itself another source of ROS production in vascular cells [38]. In addition, 12/15-LOX and its products, 12(S)-HETE and 15(S)-HETE, are implicated in the development of atherogenesis [39,40,41,42] and HF [43].

Of note, Suzuki *et al.* have recently shown in a mouse model of HF with streptozotocin-induced diabetes or db/db mice that 12/15-LOX is highly expressed in cardiac tissue and increases mitochondria- and NADPH-derived oxidative stress and thus contributes to fibrosis formation in the myocardium as well as to decreases in cardiac function [44]; treatment with the antioxidant *N*-acetyl-cysteine (NAC) or a 12/15-LOX inhibitor leads to improvements in oxidative stress in cardiac tissue as well as in cardiac function, suggesting that 12/15-LOX is implicated in the development of oxidative stress as well as in the formation of myocardial fibrosis.

### 2.2. ROS-Scavenging Systems in Diabetic Cardiovascular Disease

The ultimate effects of ROS are determined by the balance between the amount of ROS produced and the ROS-scavenging capacity of individual cells. While all cells must have some system through which to degrade and inactivate ROS, either overproduction of ROS or impaired ROS-scavenging capacity can result in ROS-induced damage [45,46]. Cells that are high oxygen consumers (e.g., phagocytic cells, red blood cells, vascular endothelial cells, cardiac muscle, liver, kidney, and brain cells) need a high expression of neutralizing enzymes to protect themselves from ROS.

#### 2.2.1. Enzymatic and Non-Enzymatic Antioxidants

Antioxidants may be categorized as enzymatic or non-enzymatic. Enzymatic antioxidants include superoxide dismutases (SOD1–3), catalase, glutathione peroxidase (GPx), and thioredoxin (Trx) (Table 1).

Three major isoforms of SOD have been described and are distinguished by their localization in different cellular compartments. SOD1 (also known as copper–zinc SOD; CuZnSOD) is found in the cytoplasm; SOD2 (MnSOD) localize to the intermembrane space of the mitochondria or mitochondrial matrix [47]; and SOD3 (extracellular SOD) is found in extracellular matrix of tissues [48]. SOD enzymes are metalloproteins that catalyzing the dismutation of ·O_2_^−^ radical to molecular oxygen and hydrogen peroxide. In doing so, they reduce the amount of intracellular ·O_2_^−^.

Catalase localizes to the peroxisome and catalyzes the conversion of H_2_O_2_ to O_2_ and H_2_O. GPx localizes to the cytosol, mitochondria and plasma membrane and serves a similar antioxidant role by converting H_2_O_2_ and lipid peroxides to water and lipid alcohols [49]. Trx, under physiological conditions, allows proteins to remain in their reduced state by countering oxidation-induced damage [50,51]. Trx induces the formation of a disulfide bond by reducing the oxidized cysteine residues on proteins. This disulfide bond is then further reduced by thioredoxin reductase and NADPH. Many of these enzymatic antioxidants are present in cardiomyocytes and vascular cells [52].

In addition to enzymatic antioxidants, there are several low-molecular-weight compounds that contribute to the antioxidant defense systems. These include endogenously synthesized (e.g., uric acid, bilirubin, and coenzyme Q10) as well as dietary substances (e.g., vitamin A, vitamin C, vitamin E, folic acid, *N*-acetylcysteine (NAC), melatonin) [53,54,55,56,57] (Table 1).

**Table 1:** Summary of major enzymatic and non-enzymatic antioxidants in human.

		Enzymatic	Non-Enzymatic
Antioxidants	Endogenously synthesized	Copper–zinc SOD; CuZnSOD (SOD1)	Uric acid
		MnSOD (SOD2)	Bilirubin
		Extracellular SOD (SOD3)	Glutathione (GSH)
		Catalase	Coenzyme Q10
		Glutathione peroxidase (GPx)	*N*-Acetylcysteine (NAC)
		Thioredoxin (Trx)	Melatonin
	Dietary substances		Vitamin A, Vitamin C
			Vitamin E
			Folic acid
			Flavomoid
			Polyphenol

#### 2.2.2. Endogenous Antioxidants in Diabetes

ROS are also known to affect the *in vivo* action of antioxidants in a diabetic state. In the kidneys of diabetic mice, not only are ROS increased, but also the expression of SOD, as well as total SOD activity, has been shown to be decreased. Similarly, SOD expression in diabetic mouse models is reduced in multiple tissues, including heart, brain, kidney and liver [58,59,60]. Additionally, DM patients show reduced SOD, catalase and GPx activity, due to excessive glycation [61]. Moreover, cardiac-specific expression of GPx levels are reduced in diabetes [62,63]. High glucose exposure has also been shown to directly suppress Trx activity *in vitro* and leads to an excessive injury response in an ischemia-reperfusion injury model. This suggests that Trx may have a cardioprotective role in DM [64]. This is consistent with suggested function of Trx as a regulator of cardiac hypertrophy.

## 3. Diabetic Heart

### 3.1. Diabetic Cardiomyopathy

Diabetic cardiomyopathy has been defined as ventricular dysfunction that occurs in diabetic patients independent of a recognized cause, such as coronary artery disease (CAD) or hypertension [65,66]. The hallmark characteristic of diabetic cardiomyopathy is a subclinical phase associated with cellular structural abnormalities leading initially to diastolic dysfunction, later to systolic dysfunction, and eventually to heart failure.

Diabetes leads to cardiac structural and functional disturbances in the myocardium. It is widely recognized that cardiomyocyte hypertrophy, cardiac inflammation, fibrosis, increased apoptosis, and metabolic abnormalities are present in diabetic cardiomyopathy [44,67,68,69,70]. ROS has also been implicated in all stages of development of heart failure (HF), from cardiac hypertrophy to fibrosis, contractile dysfunction, and failure [71].

### 3.2. Involvement of ROS in Various Disease States

#### 3.2.1. Cardiac Inflammation and Fibrosis in the Diabetic Heart

Inflammation has an important role in the pathogenesis and progression of many forms of cardiovascular disease. Chronic inflammatory process induced by pro-inflammatory cytokines and chemokines may also contribute to the pathogenesis of diabetic cardiomyopathy [72,73]. Indeed, concentrations of inflammatory cytokines, such as TNF-α and IL-6, are increased in the serum of diabetic patients, suggesting a link between this chronic inflammation and diastolic dysfunction [74].

TNF-α is an important molecule that triggers inflammation and cell injury in the heart [75], ultimately inducing cardiac fibrosis as a consequence [76,77]. Suppression of TNF-α has been shown to improve diabetic cardiomyopathy, reduce cardiac fibrosis, and enhance cardiac function [67,78,79]. NF-κB has also been implicated as a key mediator of the inflammatory process in the diabetic heart [80,81]. NF-κB is a major transcription factor that controls the expression of many genes including pro-inflammatory, pro-fibrotic and hypertrophy-related genes [74,80].

**Figure 4:**
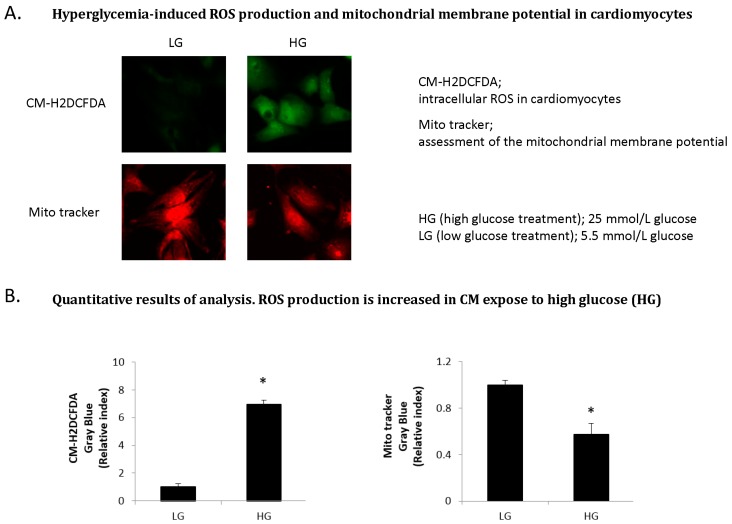

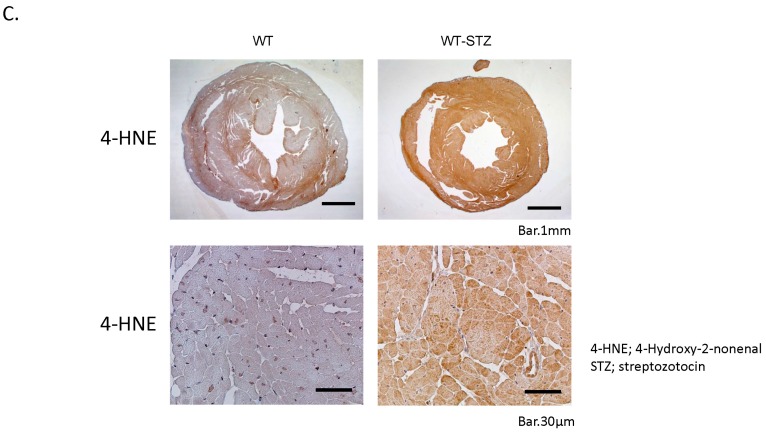
ROS production and oxidative stress in the diabetic heart *in vitro* and *in vivo*. (Copyright 2015 American Diabetes Association from [44]. Reprinted with permission from The American Diabetes Association). (**A**) Hyperglycemia-induced ROS production and mitochondrial membrane potential in cardiomyocytes. Intracellular ROS level is increased in cardiomyocytes (CM) expose to high glucose (HG) by using chloromethyl-2,7-dichlorodihydro-fluorescein diacetate (CM–H2DCFDA). There was loss of mitochondrial membrane potential (ΔΨ_m_) in CM expose to high glucose as indicated by a decrease in the fluorescence intensity assessed using Mito Tracker red; (**B**) Quantitative results of analysis. ROS production is increased in CM expose to high glucose (HG). CM-H2DCFDA, intracellular ROS in cardiomyocytes. Mito tracker, assessment of the mitochondrial membrane potential. HG (high glucose treatment), 25 mmol/L glucose; LG (low glucose treatment), 5.5 mmol/L glucose. * *p* < 0.05 *vs.* LG. Error bars indicate s.e.m. *n* = 4–6; (**C**) Cardiac oxidative stress in the diabetic heart. Immunohistological staining (brown) of 4-hydroxy-2-nonenal (4-HNE) in the hearts of wild-type (WT), wild-STZ (WT-STZ) mice. **Upper** panel is 20×, **lower** panel is 400×; Scale bar, 1 mm and 30 μm, respectively. Cardiac 4-HNE, a major marker of oxidative stress, is up-regulated in myocardium in WT-STZ heart compared to WT heart.

In agreement with these findings, Suzuki and colleagues showed that expression of TNF-α, NF-κB and collagen markers were elevated and accompanied by an increase of oxidative stress in the STZ-induced diabetic heart [44]. Intracellular ROS levels in cardiomyocytes were increased under high glucose condition by fluorimetry *in vitro*. In addition, there was loss of mitochondrial membrane potential (ΔΨm) as indicated by a decrease in the fluorescence intensity in cardiomyocytes under high glucose condition (Figure 4A,B). Moreover, expression of cardiac 4-hydroxy-2-nonenal (4-HNE), a major marker of oxidative stress, was up-regulated in STZ-induced diabetic heart (Figure 4C).

Further studies have suggested that oxidative stress is an important regulator of not only inflammation but also of pro-fibrotic processes in the heart [82,83]. Increased expression of TNF-α, NF-κB and collagen factors in diabetic heart was significantly inhibited by treatment of antioxidant (NAC). Consequently, hyperglycemia-induced cardiac fibrosis is also ameliorated by treatment with antioxidant [44]. These results indicate that cardiac oxidative stress has a major role in promoting cardiac inflammation and fibrosis in the diabetic heart. Therefore, reduction of chronic inflammation and improvement of redox balance may have important implications for the treatment and prevention of cardiac fibrosis in the diabetic heart. Indeed, therapeutic approaches targeting chronic inflammation pathways (such as IL-1 and NF-κB inhibitors) and other immunosuppressive agents are currently being evaluated in diabetes [84,85].

#### 3.2.2. Cardiac Hypertrophy and Apoptosis in the Diabetic Heart

Left ventricular hypertrophy (LVH) is a hallmark in the morphologic manifestation of diabetic cardiomyopathy, generally representing a more advanced stage of disease. Echocardiographic evidence revealed that LVH is a common structural and functional alternation in diabetic patients even in the absence of coronary artery disease or hypertension [86]. Although LVH is frequently associated with increased afterload in diabetic patients with hypertension [87], it can also occur independent of pressure-overload [86].

In the diabetic state, neurohormonal activation is seen both at the systemic and the tissue levels, and includes up-regulation of RAAS, ET-1, and the sympathetic nervous system [88,89,90]. Enhanced activity of local RAAS induces functional abnormalities in the diabetic heart. High glucose concentrations can increase RAAS activation and production of AII in cardiomyocytes and cardiac fibroblasts [91,92]. Local AII production in the diabetic heart is significantly higher than the serum levels of the peptide [92].

**Figure 5:**
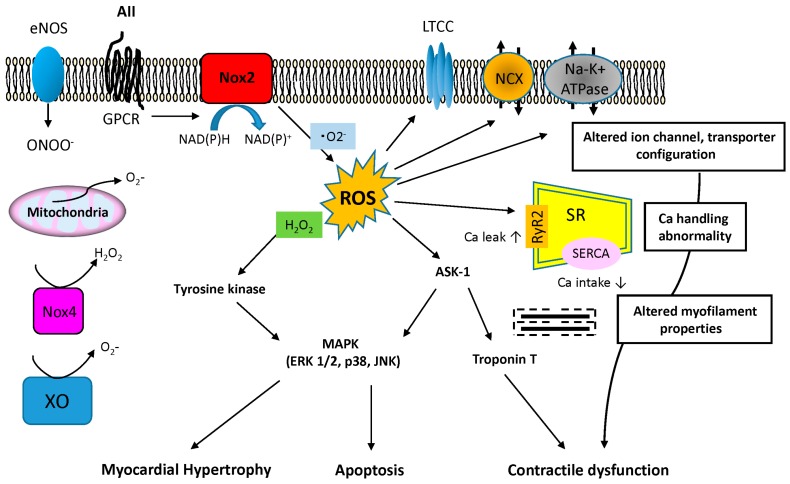
AII-associated ROS pathway and alternation of the structure and function in cardiomyocyte. AII involves activation of ·O^2^^−^ production by NADPH oxidase NOX2. AII induces cardiac hypertrophy and apoptosis via G-protein-linked pathway that involves ROS-related activation of several downstream signals, including MAPKs (ERK 1/2, p38, JNK). ASK1 is also activated by ROS and in turn activates p38, JNK and induces cardiac hypertrophy and apoptosis. ASK1 may promote troponin T phosphorylation and implicate in contractile dysfunction. AII-associated ROS pathway may influence the alternation of structure and function of excitation-contraction coupling and ionic homeostasis, including LTCC, NCX, Na–K^+^ ATPase and Ca handling. The potential effects of dysregulated ion channel, transporter and calcium in SR are shown. G-protein receptor (GPCR), l type Ca^2+^ channels (LTCC), Sodium/calcium exchanger (NCX), Sarcoplasmic reticulum (SR), Sarcoplasmic reticulum calcium calcium ATPase (SERCA), Ryanodine receptor (RyR2), Xanthine oxidase (XO).

Various stimuli are known to induce myocardial hypertrophy, myocardial fibrosis and apoptosis including neurohormonal factors and cytokines, such as AII, ET-1, TNF-α activation of redox-sensitive protein kinases and mechanical stretch [93,94]. ROS are reported to activate directly or indirectly various pathways located downstream of the hypertrophic signaling pathways, which include PKC, p38MAPK, JNK, ERK1/2, ASK-1, PI3K, Akt, NFκ-B and calcineurin [95]. AII has received particular attention as a way to stimulate membrane NADPH oxidase via G-protein coupled receptors thus inducing ·O_2_^−^ production. Subsequently, various downstream signals are activated, including the MAPK, to induce cardiac hypertrophy (Figure 5).

Using fluorescent probes in cultured cardiomyocytes, Nakamura *et al.* demonstrated that cell hypertrophy and ROS expression are increased in AII- and TNF-α-concentration-dependent fashion and that myocardial hypertrophy is suppressed with the use of antioxidants [75]. In addition to reducing myocardial ·O_2_^−^ production, antioxidants such as probucol, tempol and N-acetylcysteine (NAC) have also been shown to have other beneficial effects including prevention of Ang II-induced protein synthesis and ANP expression [96]. These studies suggested that NAPDH oxidase-dependent ROS generation plays a pivotal role in the pathophysiology and progression of cardiomyocyte hypertrophy.

Numerous experimental studies demonstrate that ROS are involved in cardiomyocyte apoptosis, an important contributing factor to decreased cardiac function in myocardial hypertrophy, ischemia and HF. The triggering of apoptosis by ROS may be concentration dependent. Low concentrations of H_2_O_2_ activate ERK1/2 MAPK, leading to cardiomyocyte hypertrophy; however, higher levels of H_2_O_2_ induce JNK, p38MAPK, and Akt in addition to the ERK1/2 MAPK pathway, and thereby promote cardiomyocyte apoptosis [97]. In addition, diethyldithiocarbamade (DDC), a Cu/Zn-SOD inhibitor, is reported to induce hypertrophy at low concentrations but promote apoptosis at higher concentrations in cultured cardiomyocytes [98]. Cardiomyocyte apoptosis plays an important role in the development of diabetic cardiomyopathy as well and the degree of cardiomyocyte apoptosis has been reported to be correlated with blood glucose levels [99,100]. In particular, increased myocyte apoptosis is implicated in the transition from a compensated to a decompensated hypertrophic state in the diabetic heart [100]. This pro-apoptotic effect of hyperglycemia is accounted for in part by p53 glycosylation and phosphorylation and excessive AII synthesis [101].

#### 3.2.3. Heart Failure and Metabolic Abnormalities in Diabetic Heart

As noted earlier, ROS-induced mitochondrial dysfunction is of particular interest in the development of HF [102,103]. While the mitochondrial respiratory chain (MRC) enzyme complexes consisting of mtDNA-encoded and nDNA-encoded subunits are required for mitochondrial oxidative phosphorylation, Ide *et al.* demonstrated in a canine model of tachyarrhythmia-induced HF that increased intra-mitochondrial ROS in the failing myocardium induces dysfunction in the adjacent mitochondrial membrane and mtDNA, resulting in impaired enzymatic activity of the MRC complex I. Moreover, dysfunction in the mitochondrial electron transfer system leads to ROS leakage and increased ROS production, thus forming a vicious cycle [19,104].

In addition to mitochondrial alterations, abnormalities in surface membrane ion channels and sarcomere-related proteins can contribute to cardiac dysfunction (Figure 5). More specifically Ca^2+^ signaling is essential for cardiomyocyte contractile function and relaxation. An important component of the Ca^2+^ signaling pathway is SERCA2a, which initiates cardiomyocyte relaxation by sequestering Ca^2+^. It is reported that ROS target the l-type Ca channels essential for calcium handling and inhibit Ca^2+^ inflow [105] and that ROS reduce SERCA2a activation [106]. As such, ROS-induced dysfunction of the sarcoplasmic reticulum can lead to dramatic intracellular calcium accumulation, diminished contractility, and arrhythmias [107]. Moreover, ROS generation can also alter the function of cardiac sodium channels, potassium channels, and ion exchangers, such as Na/Ca exchanger [107,108,109].

ROS can also decrease the calcium sensitivity of the myofilaments leading to further contractile dysfunction. The redox-sensitive kinase ASK1 has been reported to promote troponin T phosphorylation *in vitro* and *in vivo*, thus decreasing myocardial contractility (Figure 5) [107,110]. Moreover, ASK1 also activates p38 MAPK and JNK signaling, promoting apoptosis [111]. Indeed, overexpression of ASK-1 induces apoptosis in cardiomyocytes while ASK1-KO mice are protected from pressure overload-induced cardiomyocyte apoptosis and contractile dysfunction [112,113].

## 4. Diabetic Vascular Disease

The vascular complications of diabetes are conventionally divided into macrovascular and microvascular categories. While microvascular complications resulting in retinopathy, neuropathy, and nephropathy are important causes of morbidity and mortality in diabetes patients, we will primarily focus on the mechanistic role of ROS as mediators of macrovascular diabetic disease.

### 4.1. Endothelial Dysfunction/Atherosclerosis 

Alterations in endothelial cell function are critical to the process of atherogenesis. High glucose concentrations induce an imbalance between vaso-protective nitric oxide (NO) bioavailability and accumulation of reactive oxygen species (ROS) as an early mechanism leading to endothelial dysfunction [114].

Hyperglycemia may initiate this process by increasing superoxide anion production via the mitochondrial electron transport chain [17]. Subsequently, a chain reaction of further ROS-generating events and positive feedback mechanism is induced. For instance, superoxide anion activates protein kinase C (PKC) [17] that, in turn, may contribute to further superoxide generation [115,116]. Activation of PKC by glucose further leads to NADPH oxidase (NOX)-dependent ROS generation [117,118].

Apart from merely decreasing the (protective) bioavailability of NO, the rapid reaction of superoxide with NO also forms a highly reactive intermediate, peroxynitrite (ONOO^−^). Peroxynitrite in turn—being a strong cytotoxic oxidant—causes oxidative damage, nitration, and *S*-nitrosylation of biomolecules, such as proteins, lipids, and DNA, and thereby may be involved in the pathogenesis of virtually all major cardiovascular diseases (e.g., stroke, myocardial infarction, and heart failure) [119,120]. Moreover, peroxynitrite may uncouple eNOS (via oxidation of tetrahydrobiopterin), thus leading to further superoxide generation, resulting in a vicious cycle that contributes to enhanced NO depletion [121]. Only recently, oxidative stress-induced *S*-glutathionylation of eNOS was identified as another molecular mechanism leading to eNOS uncoupling and subsequent impaired endothelium-dependent vasodilation, which can be restored by thiol-specific reducing agents [122].

Mitochondrial production of superoxide anion also increases intracellular production of advanced glycation end products (AGEs) [17]. AGEs contribute to atherosclerosis by modifying the extracellular matrix and circulating lipoproteins, as well as binding to and activating the receptor for AGE (RAGE), which is present on many vascular cells [123]. Additionally, AGEs *per se* increase production of oxygen-derived free radicals, and RAGE activation increases intracellular enzymatic superoxide production [124,125,126].

Inflammation is another hallmark of atherosclerosis. Here, the redox-sensitive activation of NFκB under hyperglycemic conditions may serve as a central hub promoting a pro-inflammatory milieu [127,128,129,130,131]. Additionally, superoxide promotes atherogenesis by oxidation of LDL. Oxidized LDL (ox LDL) enhances vascular inflammation by augmenting intimal macrophage infiltration (e.g., through up-regulation of chemotactic factors such as monocyte chemotactic protein (MCP)-1, or enhanced expression of adhesion molecules such as vascular cell adhesion molecule (VCAM)-1 and intercellular adhesion molecule (ICAM)-1), leading to subsequent foam-cell formation [132].

Finally, ROS may be instrumental in promoting VSMC proliferation that constitutes another feature of atherosclerosis and restenosis after angioplasty. Patients with diabetes have increased proliferation and migration of VSMCs into atherosclerotic lesions [133]. Mechanistically, NOX-mediated ROS production was shown to promote the pro-proliferative effects of hyperglycemia *in vitro* [134]. Additionally, high glucose-induced VSMC proliferation involves NFκB as another redox-sensitive element [131].

Taken together, there is abundant evidence for a critical role of ROS as mediators of accelerated atherosclerosis in diabetes.

### 4.2. Arterial Stiffness

Conduit arteries, such as the aorta, become stiffer with age [135,136]. However, one of the mechanisms linking diabetes to increased cardiovascular risk may be accelerated arterial stiffening that is frequently observed in diabetic patients [137]. The Hoorn study demonstrated that type II diabetes mellitus is associated with increased central arterial stiffness [138]. Importantly, arterial stiffness predicts the development of cardiovascular disease and mortality in the general population and in type 2 diabetes [139,140].

The mechanical properties of conduit arteries are largely defined by its passive (structural) stiffness that in turn is mainly determined by the extracellular matrix (ECM) components of the load-bearing medial layer [141,142]. Fibrillar collagens (type 1 and type 3) as well as elastin represent the major ECM proteins of the aorta. While elastin is a relatively inert protein, collagen is readily remodeled in response to various stimuli. ROS-mediated signaling may be critically involved in the mechanisms leading to arterial ECM remodeling and stiffness. For instance, oxidative stress increases collagen I production in smooth muscle cells [143] and fibroblasts [144]. Moreover, ROS induce the elastin degrading matrix metalloproteinases MMP-2 and MMP-9 [145,146]. Previously, we have shown that diabetic arterial stiffness develops independently of arterial hypertension in a diabetic mouse model (db/db mouse) and found that oxidative stress induced aortic medial fibrosis via an NFκB-mediated activation of the osteogenic transcription factor Runx2 in aortic VSMC [147].

The arterial wall may also stiffen due to calcification of the elastic lamellae, termed medial elastocalcinosis (MEC) [148], which is frequently found in diabetic patients [149]. In animal models of MEC there is a strong correlation between aortic calcium content and arterial stiffness [150,151]. Interestingly, medial calcification is associated with local expression of mineralization-regulating proteins that are normally expressed in osteogenesis [149]. This observation gave rise to the now widely accepted concept that vascular calcification is an active cell-driven process characterized by osteogenic differentiation of vascular cells. Indeed, VSMCs, the dominant cell type in the calcifying media, may acquire an osteogenic phenotype, expressing bone/mineralization-associated proteins (e.g., Runx2, Sox9, Msx2) [152,153] that actively regulate arterial calcification [154,155,156]. In particular, the osteogenic transcription factor Runx2 received attention in vascular biology, emerging as a marker of VSMC trans-differentiation towards an osteogenic phenotype, and contributing to the active vascular calcification process [154,157,158]. However, while Runx2 is induced in diabetic aortae via redox-sensitive mechanisms to promote vascular fibrosis (see above), Runx2 overexpression *per se* is insufficient to induce vascular calcification [147], underlining the complex, multi-factorial nature of calcification processes [149,159].

Finally, a redox-sensitive formation of advanced glycation end-products (AGEs), causing cross-linking of collagen molecules, may lead to loss of collagen elasticity and a subsequent increase in arterial stiffness [160]. Indeed, AGEs have been associated with greater stiffness in diabetic patients [161,162], and cross-link breakers have been demonstrated to decrease arterial stiffness in humans [163].

### 4.3. Abdominal Aortic Aneurysm (AAA)

An abdominal aortic aneurysm (AAA) is a permanent, localized dilatation of the abdominal aorta (beginning at the level of the diaphragm and extending to its bifurcation into the left and right common iliac arteries) that exceeds the normal diameter by 50%, or >3 cm [164]. Pathomechanistically, AAA exhibit significantly increased ROS production that may result in vascular inflammation, extensive ECM remodeling and VSMC apoptosis, all of which are critical features of AAA disease [16]. Thus, anti-oxidant interventions were found protective in the context of experimental AAA [165,166,167].

Paradoxically, diabetes, a strong inducer of vascular oxidative stress, low-grade inflammation and potent driver of vascular disease, was shown to be protective against the development of AAA [168,169]. Moreover, diabetes is also associated with a slower rate of growth of established AAAs [169]. While the underlying mechanism for this puzzling phenomenon is still unclear, we recently demonstrated that experimental AAA growth is driven by mechanical forces driven by a stiffness gradient between the AAA segment and the more compliant adjacent aorta [170]. Thus, as diabetes generally increases arterial stiffness, this may reduce segmental aortic stiffness gradients and prevent AAA formation.

## 5. Therapeutic Perspectives for Diabetic Cardiovascular Disease

A wide variety of compounds and drugs have been investigated for their antioxidant properties and potential benefits in cardiovascular disease. These include medications that have become standard-of-care such as HMG CoA reductase inhibitors (statins), angiotensin convertings enzyme inhibitors (ACE-I), angiotensin receptor blockers (ARBs), all of which have separate primary roles but also show antioxidant characteristics [171]. Given their known antioxidant capabilities, various vitamins including vitamin C and vitamin E have been the subject of active investigation in the realm of cardiovascular protection, but large randomized trials of antioxidant vitamins have been unsuccessful, and this failure might be ascribed to the nonspecific nature of such agents, which could inhibit both beneficial and detrimental effects of ROS [172].

Another old agent with future therapeutic potential is probucol, a potent antioxidant molecule, which was originally developed for industrial use, but later displayed pharmacologic properties including lipid lowering and which has been found to prevent atherosclerosis and reduce secondary cardiovascular events in various studies [173,174]. When combined with atorvastatin in acute coronary syndrome, probucol appears to lower oxidized LDL and paraoxonase-1 beyond those achieved with statin alone [175]. There is also evidence that probucol may prevent atrial remodeling in diabetes through inhibition of ROS [176].

Another promising avenue is the omega-3 polyunsaturated fatty acids (omega-3 PUFAs). PUFAs have been linked to considerable cardiovascular benefits in diabetics and heart failure patients [177,178,179]. Consistent with the above, a rat model of diabetes shows that PUFAs alter favorably the lipid metabolism in diabetic rats themselves and on the oxidation level of their offspring [180]. This fatty acid acts as an indirect anti-oxidant in vascular endothelial cells and reduced inflammation and levels of adhesion molecules, in turn diminish the risk of atherosclerosis and cardiovascular disease [181].

Targeting oxidative stress and mitochondrial dysfunction simultaneously would seem to be a logical alternative approach. Indeed, mitochondrial-targeted antioxidants, such as the ubiquinone derivative mito-Q and mitochondrial-targeted peptides have shown promise in preclinical studies [182]. Mito-Q decreased adiposity, hypercholesterolemia, and hypertriglyceridemia in mouse models of atherosclerosis and metabolic syndrome, reduced oxidative liver damage, and reduced plaque macrophage content [183]. Much interest has also been generated regarding supplementation with the antioxidant coenzyme Q-10 (CoQ10), which is an endogenous cofactor needed for mitochondrial energy production. It has been found to diminish cardiomyopathy and attenuate diastolic dysfunction and fibrosis in diabetic mouse models [184,185]. Recently, a randomized controlled trial showed a reduction in mortality in heart failure patients who received CoQ10 compared to placebo [186]. However, the results of this study have some limitations. The striking improvements in cardiovascular outcomes with CoQ10 were not associated with difference in NYHA class and levels of N-terminal pro-brain natriuretic peptide (NT-proBNP) between groups. Similarly, supplementation with vitamin E of both the CoQ10 and placebo group complicates the interpretation of the results.

Unfortunately effects of CoQ10 on metabolic profiles in diabetes and outcomes in clinical trials have been negligible to date, apart from some reduction in triglyceride levels [187]. However, these studies have been small, limiting conclusive determinations regarding its efficacy.

## 6. Conclusions

Several lines of evidence indicate that oxidative stress plays a pivotal role in the development of diabetic-induced cardiovascular disease. Hyperglycemia and metabolic abnormalities of diabetes cause the production of ROS in vascular cells and in cardiomyocytes. Diabetic oxidative stress occurs by multiple mechanisms, with prominent roles of mitochondrial dysfunction and NOX enzymes, and in cardiac and vascular dysfunction including chronic inflammation, fibrosis, apoptosis, VSMC proliferation and arterial stiffness. As such, therapeutic strategies to reduce ROS production or enhance ROS degradation should have protective effects against diabetes-induced cardiovascular disease. Up to this point, however, randomized clinical trials have yet to provide convincing evidence that antioxidant treatment has beneficial effects in human diabetic cardiovascular disease. However, this may not necessarily be regarded as a general failure of the ROS hypothesis but may simply reflect the numerous open questions regarding antioxidant treatment. Further study to understand the initiation of oxidative stress as well as its downstream effects on cellular function will likely add more insight into the underlying mechanisms of diabetes-induced cardiovascular disease and identify more specific targeted therapies.
